# A New Total Uncertainty Measure from A Perspective of Maximum Entropy Requirement

**DOI:** 10.3390/e23081061

**Published:** 2021-08-17

**Authors:** Yu Zhang, Fanghui Huang, Xinyang Deng, Wen Jiang

**Affiliations:** School of Electronics And Information, Northwestern Polytechnical University, Xi’an 710072, China; zhangyuchn@mail.nwpu.edu.cn (Y.Z.); huangfanghui@mail.nwpu.edu.cn (F.H.); xinyang.deng@nwpu.edu.cn (X.D.)

**Keywords:** total uncertainty measure, dempster-shafer theory, maximum entropy, non-specificity

## Abstract

The Dempster-Shafer theory (DST) is an information fusion framework and widely used in many fields. However, the uncertainty measure of a basic probability assignment (BPA) is still an open issue in DST. There are many methods to quantify the uncertainty of BPAs. However, the existing methods have some limitations. In this paper, a new total uncertainty measure from a perspective of maximum entropy requirement is proposed. The proposed method can measure both dissonance and non-specificity in BPA, which includes two components. The first component is consistent with Yager’s dissonance measure. The second component is the non-specificity measurement with different functions. We also prove the desirable properties of the proposed method. Besides, numerical examples and applications are provided to illustrate the effectiveness of the proposed total uncertainty measure.

## 1. Introduction

With the development of sensor technology, it has become a trend for complex systems to be equipped with multiple sensors. Compared with single-sensor monitoring, multi-sensor monitoring could have higher reliability. Obviously, it is a key issue to effectively fuse the multi-sensor information. Many techniques are proposed to solve the issue, such as the Dempster-Shafer theory (DST) [1,2], Kalman filtering (KF) [3,4], fuzzy theory [5], Bayesian reasoning method [6,7], neural network [8], and so on. However, there are many uncertainties, for example, randomness and imprecision, in the real world. The treatment of uncertainty is an important aspect in information fusion theories. Among them, DST is an effective framework to deal with uncertain information. This theory was first proposed by Dempster [9] and further developed by Shafer [10]. It is widely used in fault diagnosis [11,12,13,14], decision-making [15,16], risk assessment [17], and so on. Many studies in recent years have focused on conflict resolution [18,19,20], evidence revision [21], combination rules [22,23,24,25], and information volume [26,27]. Many methods about uncertainty quantification have also been proposed [28]. However, the existing methods have some limitations, and the uncertainty measure of BPAs is still an open issue in DST.

The concept of entropy was first proposed by the German physicist Clausius in 1865. In thermodynamics, entropy is a measure of the “chaos” of a system [29]. In information theory, entropy, also known as Shannon entropy, represents a measure of the uncertainty of a random variable [30]. Besides, Ilya Prigogine proposed a famous statement: “The entropy …leads to an ordering process” [31]. Parker and Jeynes also showed from a MaxEnt standpoint that the (stupendously gigantic) entropy of the supermassive black hole at the centre of the Milky Way can account for the geometrical stability of the galaxy [32]. Among them, the Shannon entropy, verified in the past few decades, is an effective way to measure uncertainty in probability theory (PT), but its direct application in DST is inappropriate. That is because that PT describes the probability of the occurrence of singletons, while the evidence theory is based on the theory of non-additive probabilities, which can represent the possibility of the occurrence of propositions with multiple elements [33].

Based on the above analysis, many scholars have proposed different entropy-like measures to quantify the uncertainty of a body of evidences (BOEs) in DST. For instance, Nguyen proposed a belief entropy based on the original basic probability assignment (BPA) [34]. Dubois and Prade proposed a weighted Hartley entropy for measuring the non-specificity of BPAs [35]. In addition, many other belief entropies have been proposed, including Höhle’s entropy [36], Yager’s dissonance measure [37], Klir and Ramer’s discord measure [38], Klir and Parviz’s strife measure [39], Jousselme’s ambiguity measure (AM) [40], Deng entropy [41], Yang and Han’s measure [42], the aggregated uncertainty measure (AU) [43], Wang and Song’s measure (SU) [44], Jirousek and Shenoy’s entropy (JS) [45], Deng’s measure [46], and so on [47,48,49]. These methods can effectively measure the uncertainty of BOEs in some cases, and satisfy some desirable properties of uncertainty quantification in DST [50]. Intuitively, when the system is completely unknown, that is, $m\left(\Omega \right)=1$, Ω represents a frame of discernment (FOD), the uncertainty of evidence is the greatest, which is called the maximum entropy property. Some of the existing methods do not support this property. However, we think that maximum entropy should be a property that must be satisfied.

Motivated by the above discussions, a new total uncertainty measure from a perspective of maximum entropy requirement is proposed. The proposed method can measure both dissonance conflict and non-specificity in BPA, which includes two components. The first component is consistent with Yager’s dissonance measure. The second component is the non-specificity with different functions. We also prove the majority of the desired properties of the proposed method, such as non-negativity, monotonicity, probability consistency, and so on. The main contributions are summarized as follows.

- We propose a new total uncertainty measure from the perspective of the maximum entropy requirement to quantify the uncertainty of BPAs in DST. Besides, properties of the proposed method have also been proved, such as non-negativity, monotonicity, maximum entropy, and so on.
- We conduct some numerical examples to evaluate the effectiveness of our proposed method. The simulation results indicated that our proposed total uncertainty measure could be degraded to Shannon entropy when BPA is a Bayesian mass function. Furthermore, the proposed entropy could effectively deal with the redundant information of the focal element.

The remainder of this paper is organized as follows. Section 1 reviews the related concepts and works. Some preliminaries are introduced in Section 2. In Section 3, we illustrate the proposed method in detail. Simulation results and discussion compared with other methods are presented in Section 4. In Section 5, we give an application in feature evaluation for pattern classification based on the Iris dataset. Section 6 is a conclusion of the paper.

## 2. Preliminaries

Some basic concepts and existing methods are briefly introduced in this section.

### 2.1. Dempster-Shafer Theory

The Dempster-Shafer theory, proposed by Dempster [9] and expanded by Shafer [10], is a mathematical theory of multi-source information. This method can effectively cope with uncertainty. It is widely used in target identification, fusion decision, and so on. Some definitions about this theory are as follows.

- *Frame*ofDiscernment(FOD). If $\Theta =\left\{\theta_{1},\theta_{2},\cdots ,\theta_{r}\right\}$ is a finite complete set of *r* mutually exclusive elements, it is called a frame of discernment [9,10,51].
- BasicProbabilityAssignment(BPA). Set Θ is a FOD, and its power set constitutes a set of propositions. If a function $m:2^{\Theta }\to \left[0,1\right]$ satisfies the following formula [9,10,52]:
  (1) $$\left\{\begin{array}{c} m\left(\emptyset \right)=0\\ \sum_{S}m\left(S\right)=1 \end{array}\right.,$$
  the mass function *m* is a BPA. In this definition, $m\left(S\right)$ is the basic probability number of proposition *S*, and indicates the belief assigned to *S*.
- DempsterCombinationRule(DCR). Let $m_{1}$ and $m_{2}$ be two BPAs, and then the Dempster combination rule is as follows [9]:
  (2) $$\left\{\begin{array}{c} m\left(\emptyset \right)=0\\ m\left(C\right)=\frac{\sum_{S\cap B=C}m_{1}\left(S\right)m_{2}\left(B\right)}{1-k},\;C\neq \emptyset \end{array}\right.$$
  where $k=\sum_{S\cap B=\emptyset }m_{1}\left(S\right)m_{2}\left(B\right)$.

### 2.2. Belief and Plausibility Function

Let *m* be a BPA on FOD Θ, if $Bel:2^{\Theta }\to \left[0,1\right]$ statisfies [9]:

(3) $$Bel\left(S\right)=\sum_{B\subseteq S}m\left(B\right),\;S\in 2^{\Theta }\;\;$$

then, $Bel\left(S\right)$ is called the belief measure of proposition *S*.

$Pl\left(S\right)$ is the plausibility function that is defined as follows:

(4) $$Pl\left(S\right)=1-Bel\left(\bar{S}\right)=\sum_{B\cap S=\emptyset }m\left(B\right)$$

$Pl\left(S\right)$ is the degree to which you do not disagree with proposition *S*.

### 2.3. Shannon Entropy

Let X be a sample space with possible values $\left\{x_{1},x_{2},\cdots ,x_{n}\right\}$, then, the Shannon entropy is defined as [30]:

(5) $$H_{s}=\sum_{x_{i}\in X}p\left(x_{i}\right)\log_{2}\frac{1}{p\left(x_{i}\right)},$$

where $p\left(x_{i}\right)$ is the probability of $x_{i}$. It also satisfies $\sum_{x_{i}\in X}p\left(x_{i}\right)=1$.

### 2.4. Some Existing Entropies in DST

In the information theory, Shannon entropy has been widely used, but it has an inherent limitation of handling the uncertainty in DST. Nonetheless, the idea of Shannon entropy still plays a crucial role in guiding the uncertainty measurement of BPA. Many scholars proposed methods of uncertainty measurements. Let *X* be a FOD, and some existing uncertainty measures in DST are listed as follows.

**Nguyen’s entropy.**  Nguyen [34] proposed a belief entropy based on the original BPA:
  (6) $$H_{N}=\sum_{S\in 2^{X}}m\left(S\right)\log_{2}\frac{1}{m\left(S\right)}.$$

**Weighted Hartley entropy.**  Dubois and Prade [35] proposed a entropy for the non-specificity measure:
  (7) $$H_{DP}=\sum_{S\in 2^{X}}m\left(S\right)\log_{2}\left|S\right|,$$
  where $\left|S\right|$ is the cardinality of *S*.

**Aggregated uncertainty measure (AU).**  Harmanec and Klir [43] proposed a total uncertainty measure of non-specificity and inconsistency:
  (8) $$AU\left(m\right)=\max\left[-\sum_{x\in X}p_{x}\log_{2}\left(p_{x}\right)\right],$$
  where $p_{x}$ is defined as:
  (9) $$\left\{\begin{array}{c} p_{x}\in \left[0,1\right],\;\forall x\in X\\ \sum_{x\in X}p_{x}=1\\ \sum_{x\in S}p_{x}\in \left[Bel\left(S\right),Pl\left(S\right)\right],\;\forall S\subseteq X \end{array}\right.$$

**Yager’s entropy.**  Yager [37] proposed a dissonance measure of BPAs based on the plausibility function:
  (10) $$H_{Y}\left(m\right)=-\sum_{S\in 2^{X}}m\left(S\right)\log_{2}Pl\left(S\right),$$
  where $Pl\left(S\right)$ is the plausibility measurement of *S* in *m*.

**Deng entropy.**  Deng [41] proposed a new uncertainty measurement method, namely, ”Deng entropy”. It is defined as:
  (11) $$\begin{array}{c} H_{Deng}\left(m\right)=-\sum_{S\in 2^{X}}m\left(S\right)\log_{2}\frac{m\left(S\right)}{2^{\left|S\right|}-1}\\ =\sum_{S\in 2^{X}}m\left(S\right)\log_{2}\left[\frac{1}{m\left(S\right)}\right]+\sum_{S\in 2^{X}}m\left(S\right)\log_{2}\left[2^{\left|S\right|}-1\right]. \end{array}$$

**Höhle entropy.**  Höhle [36] proposed a belief entropy based on a belief function, which is defined as:
  (12) $$H_{c}\left(m\right)=-\sum_{S\in 2^{X}}m\left(S\right)\log_{2}Bel\left(S\right),$$
  where $Bel\left(S\right)$ is the belief measurement of *S*.

**Yang and Han’s measure (**$TU^{I}$**).**  Yang and Han [42] defined a total uncertainty measurement. The formula is defined as:
  (13) $$TU^{I}\left(m\right)=1-\frac{\sqrt{3}}{n}\sum_{x\in X}d^{I}\left(\left[Bel\left(x\right),Pl\left(x\right)\right],\left[0,1\right]\right),$$
  where *n* is the elements number of FOD *X*. The interval distance is defined as:
  (14) $$d^{I}\left(\left[c_{1},d_{1}\right],\left[c_{2},d_{2}\right]\right)=\sqrt{{\left[\frac{c_{1}+d_{1}}{2}-\frac{c_{2}+d_{2}}{2}\right]}^{2}+\frac{1}{3}{\left[\frac{d_{1}-c_{1}}{2}-\frac{d_{2}-c_{2}}{2}\right]}^{2}}.$$

**Deng’s measure (**$TU_{E}^{I}$**).**  In addition, Deng et al. [46] proposed an improved total uncertainty measurement method based on belief intervals:
  (15) $$TU_{E}^{I}\left(m\right)=\frac{\sum_{x\in X}\left[1-d_{E}^{I}\left(\left[Bel\left(x\right),Pl\left(x\right)\right],\left[0,1\right]\right)\right]}{\left|X\right|},$$
  where $d_{E}^{I}\left(\cdot \right)$ indicates the Euclidean distance between two interval numbers $\left[Bel\left(x\right),Pl\left(x\right)\right]$ and $\left[0,1\right]$.

## 3. Proposed Uncertainty Measure in DST

### 3.1. The Proposed Method

In this section, a new total uncertainty measure of BPAs in DST is proposed, which is from a perspective of maximum entropy requirement. It can quantify the total uncertainty of BPAs, including conflict and non-specificity. As for the conflict measure of BPAs, we utilize the method of Yager’s dissonance entropy [37]. As for the non-specificity measure of BPAs, $H_{n-s}\left(m\right)$, we think that $H_{n-s}\left(m\right)$ should be consistent with the maximum entropy requirement. For example, the uncertainty of BPA $m\left(S\right)=1$ defined on FOD *X*, S⊂X, should equal to the uncertainty of vacuous BPA $m^{\prime }\left(\Omega \right)=1$, where Ω is the FOD and Ω=S. Furthermore, the uncertainty of $m\left(S\right)=a$ should hence be a function of *a* and the uncertainty degree of BPA $m\left(S\right)=1$, but not measured by the weighted Hartley entropy.

Based on the above idea, the proposed new total uncertainty measure is defined as follows:

(16) $$\begin{array}{ccc} H\left(m\right) & = & \sum_{S\in 2^{X}}m\left(S\right)\log_{2}\frac{1}{Pl\left(S\right)}+\sum_{S\in 2^{X}}m\left(S\right)Q_{S}\\ & = & \sum_{S\in 2^{X}}m\left(S\right)\log_{2}\frac{2^{Q_{S}}}{Pl\left(S\right)}, \end{array}$$

where *X* is the FOD, and $Q_{S}$ represents the maximum entropy in *S*, that is, the uncertainty of $m\left(S\right)=1$. Logically, for S⊆X, $Q_{S}$ is a monotonically increasing function of the cardinality of *S*, that is, $Q_{X}\geq Q_{S}$. In addition, when the BPA is a Bayesian mass function, we expect the new entropy to be degraded to Shannon’s entropy. Therefore, $Q_{S}=0$ when $\left|S\right|=1$. In summary, $Q_{S}$ is a function $Q_{S}:\left|S\right|\to R$, satisfying (i) $Q_{S}=0\;if\;\left|S\right|=1$; and (ii) $\frac{dQ_{S}}{d\left|S\right|}\geq 0$.

### 3.2. Properties of the Proposed Method

Similar to the probability theory (PT), there are some properties which need to be satisfied for the uncertainty measurement in DST, such as probability consistency, additivity, non-negativity, and so forth. The properties analysis of the proposed entropy is explored as follows.

**Property** **1**  (Non-negativity). *$H\left(m\right)\geq 0$, the equation holds if, and only if $m\left(\left\{x\right\}\right)=1$ and x∈X.*

**Proof.**  Given $X=\left\{x_{1},x_{2},\cdots ,x_{n}\right\}$, for any $S\in 2^{X}$,

$$0\leq m\left(S\right)\leq 1,$$

$$\left|S\right|>0,$$

$$Q_{A}\geq 0,$$

then,

$$\sum_{S\in 2^{X}}m\left(S\right)\log_{2}\frac{1}{Pl\left(S\right)}\geq 0,$$

$$\sum_{S\in 2^{X}}m\left(S\right)Q_{S}\geq 0,$$

hence, $H\left(m\right)\geq 0$. If $H\left(m\right)=0$, there must be $m\left(S\right)=0$ or $\frac{2^{Q_{S}}}{Pl\left(S\right)}=1$, that is, $m\left(\left\{x_{i}\right\}\right)=1$ and $x_{i}\in X$. □

**Property** **2**  (Set Monotonicity). *Let $m\left(X_{1}\right)=1$ be a vacuous BPA on FOD $X_{1}$, and $m\left(X_{2}\right)=1$ be also a vacuous BPA on FOD $X_{2}$, if $\left|X_{1}\right|<\left|X_{2}\right|$, then $H\left(m_{1}\right)<H\left(m_{2}\right)$.*

**Proof.**  For any vacuous BPA $m\left(X\right)=1$, there is $Pl\left(X\right)=1$, then

$$\begin{array}{ccc} H\left(m\right) & = & m\left(X\right)Q_{X}+m\left(X\right)\cdot \log_{2}\frac{1}{Pl\left(X\right)}\\ & = & m\left(X\right)Q_{X}\\ & = & Q_{X}. \end{array}$$

From the analysis in Section 3.1, it can be obtained that $Q_{S}$ is a monotonically increasing function of $\left|X\right|$, and we have $Q_{X_{1}}<Q_{X_{2}}$ if $\left|X_{1}\right|<\left|X_{2}\right|$. Hence, the proposed method satisfies the set monotonicity property. □

**Property** **3**  (Maximum entropy). *For all BPAs defined on a FOD X, the vacuous BPA $m\left(X\right)=1$ has the most uncertainty.*

**Proof.**  Let *m* be a BPA on FOD X. According to the analysis of Section 3.1, $Q_{S}$ is a monotonically increasing function of the cardinality of *S*. Therefore, the proposed method obviously satisfies the maximum entropy property. □

**Property** **4**  (Probability consistency). *For a Bayesian BPA defined on FOD X, its uncertainty equals to the form of Shannon entropy $H\left(m\right)=\sum_{S\in X}m\left(S\right)\log_{2}\frac{1}{m\left(S\right)}.$*

**Proof.**  If *m* is a Bayesian BPA, then $Pl\left(S\right)=m\left(S\right)$, hence,

$$\begin{array}{ccc} H\left(m\right) & = & \sum_{S\in X}m\left(S\right)\log_{2}\frac{1}{Pl\left(S\right)}+\sum_{S\in X}m\left(S\right)Q_{S}\\ & = & \sum_{S\in X}m\left(S\right)\log_{2}\frac{1}{m\left(S\right)}+\sum_{S\in X}m\left(S\right)Q_{S}\;\left(\begin{array}{c} since\;Q_{S}=0\;\\ if\;\left|S\right|=1 \end{array}\right)\\ & = & \sum_{S\in X}m\left(S\right)\log_{2}\frac{1}{m\left(S\right)}.\; \end{array}$$

Therewith, the proposed method satisfies the property of probability consistency. □

**Property** **5**  (Range). *The range of the proposed entropy is $\left[0,Q_{X}\right]$, where $Q_{X}$ is a function of $\left|X\right|$.*

**Property** **6**  (Non-Additivity). *Let $m_{X}$ and $m_{Y}$ be two BPAs that are defined on FOD X and Y, respectively. Then, $H\left(m_{X}⊕m_{Y}\right)\neq H\left(m_{X}\right)+H\left(m_{Y}\right)$.*

**Proof.**  Let *Z* be the Cartesian product space Z=X×Y, where x∈X and y∈Y, $Z=\{z_{11},z_{12},\cdots ,z_{lk}\}$ is the corresponding joint focal element on *Z*, $z_{ij}=\left\{x_{i},y_{j}\right\}$, and $\left(m_{X}⊕m_{Y}\right)\left(A\times B\right)=m_{X}\left(A\right)m_{Y}\left(B\right)$. Then, the new entropy of $m_{X}⊕m_{Y}$ is:

$$\begin{array}{ccc} H\left(m_{X}⊕m_{Y}\right) & = & \sum_{z\in 2^{Z}}m\left(z\right)Q_{z}+\sum_{z\in 2^{Z}}m\left(z\right)\log_{2}\frac{1}{Pl\left(z\right)}\\ & = & \sum_{z\in 2^{Z}}m\left(z\right)\log_{2}\frac{2^{Q_{z}}}{Pl\left(z\right)}\\ & \neq & \sum_{S\in 2^{X}}\sum_{B\in 2^{Y}}m_{X}\left(S\right)m_{Y}\left(B\right)\log_{2}\frac{2^{Q_{S}Q_{B}}}{Pl\left(S\right)Pl\left(B\right)}\\ & = & \sum_{S\in 2^{X}}m_{X}\left(S\right)\log_{2}\frac{2^{Q_{S}}}{Pl\left(S\right)}+\sum_{B\in 2^{Y}}m_{Y}\left(B\right)\log_{2}\frac{2^{Q_{B}}}{Pl\left(B\right)}\\ & = & H\left(m_{X}\right)+H\left(m_{Y}\right). \end{array}$$

Therefore, the proposed method does not satisfy the additivity property. □

**Property** **7**  (Generalized Set Consistency). *When $m\left(S\right)=1$, S is any subset of a FOD, $H\left(m\right)=f\left(\left|S\right|\right)$, where f is a monotonically increasing function of $\left|S\right|$.*

**Proof.**  *Assuming m is a BPA defined on FOD X, for $S\in 2^{X}$ and $m\left(S\right)=1$, the uncertainty measurement based on the proposed entropy is:*

$$\begin{array}{ccc} H\left(m\right) & = & \sum_{S\in X}m\left(S\right)Q_{S}+\sum_{S\in X}m\left(S\right)\log_{2}\frac{1}{Pl\left(S\right)}\\ & = & m\left(S\right)Q_{S}\\ & = & Q_{S}. \end{array}$$

*Therefore, the proposed entropy satisfies the property of generalized set consistency if, and only if $Q_{S}=\log_{2}\left|S\right|$.*

## 4. Numerical Examples

In this section, we give three different forms of $Q_{S}$. Some numerical examples are given to verify the rationality and effectiveness of the proposed method.

**Case 1 (**$Q_{S}^{1}$**).**  According to [45], the maximum entropy is $2\log_{2}\left|X\right|$, where *X* is a FOD. In this paper, $Q_{S}:S\to R$ represents the maximum entropy in *S*. Hence, based on the above analysis, one function form of $Q_{S}$ can be defined as:
  $$Q_{S}^{1}=2\log_{2}\left|S\right|.$$

**Case 2 (**$Q_{S}^{2}$**).**  According to [26], the maximum Deng entropy is $\log_{2}\sum_{S\in 2^{X}}\left(2^{\left|S\right|}-1\right)$. Theoretically, $Q_{S}^{2}$ should be $\log_{2}\sum_{B\in 2^{S}}\left(2^{\left|B\right|}-1\right)$. However, the Deng entropy’s maximum uncertainty is obtained at $m\left(S\right)=\frac{\left(2^{\left|S\right|}-1\right)}{\sum_{S\in 2^{X}}\left(2^{\left|S\right|}-1\right)}$, which is inconsistent with our idea. In this paper, we think that the uncertainty of $m\left(S\right)=a$ should be a function of *a* and uncertainty degree of BPA $m\left(S\right)=1$. Hence, for any $B\in 2^{S}$, there is only one situation, B=S. Therefore, another function form of $Q_{S}$ can be defined as:
  $$Q_{S}^{2}=\log_{2}\left(2^{\left|S\right|}-1\right).$$

**Case 3 (**$Q_{S}^{3}$**).**  According to [44,46], the maximum entropy is $\left|X\right|$, where *X* is a FOD. Similarly, the third function form of $Q_{S}$ can be defined as:
  $$Q_{S}^{3}=\left\{\begin{array}{c} 0,\;\;\;\;\left|S\right|=1\\ \left|S\right|,\;\;\left|S\right|>1 \end{array}\right.$$

Then, the proposed entropy could be written as follows:

(17) $$\begin{array}{ccc} H^{1}\left(m\right) & = & \sum_{S\in 2^{X}}m\left(S\right)\log_{2}\frac{1}{Pl\left(S\right)}+\sum_{S\in 2^{X}}m\left(S\right)\cdot 2\log_{2}\left|S\right|\\ & = & \sum_{S\in 2^{X}}m\left(S\right)\log_{2}\frac{2^{2\log_{2}\left|S\right|}}{Pl\left(S\right)}. \end{array}$$

(18) $$\begin{array}{ccc} H^{2}\left(m\right) & = & \sum_{S\in 2^{X}}m\left(S\right)\log_{2}\frac{1}{Pl\left(S\right)}+\sum_{S\in 2^{X}}m\left(S\right)\log_{2}\left(2^{\left|S\right|}-1\right)\\ & = & \sum_{S\in 2^{X}}m\left(S\right)\log_{2}\frac{2^{\left|S\right|}-1}{Pl\left(S\right)} \end{array}$$

(19) $$\begin{array}{ccc} H^{3}\left(m\right) & = & \left\{\begin{array}{c} \sum_{S\in 2^{X}}m\left(S\right)\log_{2}\frac{1}{Pl\left(S\right)},\;\;\;\;\;\left|S\right|=1\\ \sum_{S\in 2^{X}}m\left(S\right)\log_{2}\frac{1}{Pl\left(S\right)}+\sum_{S\in 2^{X}}m\left(S\right)\cdot \left|S\right|,\;\;\;\left|S\right|>1 \end{array}\right.\\ & = & \left\{\begin{array}{c} \sum_{S\in 2^{X}}m\left(S\right)\log_{2}\frac{1}{Pl\left(S\right)},\;\;\;\;\;\left|S\right|=1\\ \sum_{S\in 2^{X}}m\left(S\right)\log_{2}\frac{2^{\left|S\right|}}{Pl\left(S\right)},\;\;\;\;\;\left|S\right|>1 \end{array}\right. \end{array}$$

### 4.1. Example 1

This example is adapted from [53]. Let the FOD be $X=\left\{x_{1},x_{2},\cdots ,x_{n}\right\}$. We give a BPA as mxi=11nn. Then, we calculate the uncertainty of this BPA when *n* changes.

According to the definition and the desired properties of the entropy, it can be inferred that as *n* increases, the uncertainty of this BPA increases. In addition, when the BPA is a Bayesian mass function, the uncertainty of the BPA should be consistent with Shannon entropy.

**Figure 1:**
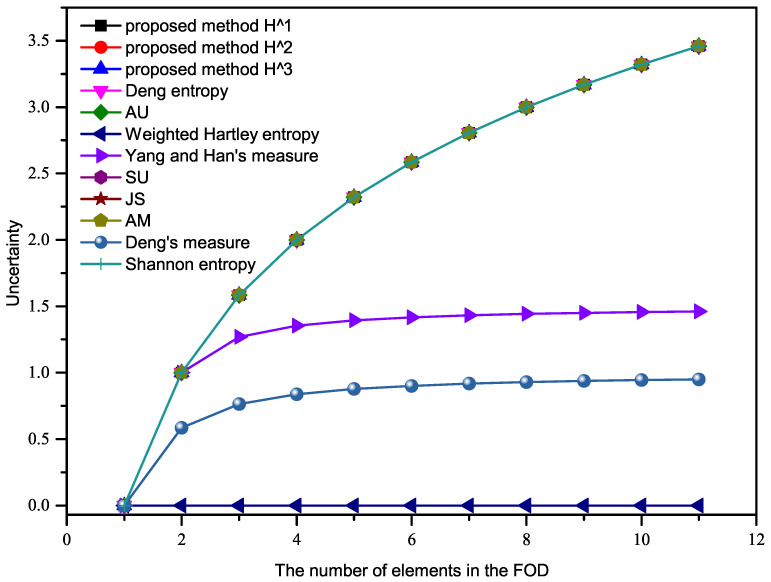
Comparison results of Example 1.

We calculate the uncertainty of this BPA in this example based on the proposed method and some existing methods, as shown in Figure 1.

For this example, all the methods gave exactly the same result as the Deng Entropy (except the weighted Hartley entropy, and the two different “measures” of Deng, as well as Yang and Han). In this paper, the “Deng entropy“ was proposed by Deng to measure the uncertainty of BPAs in 2016, while “Deng’s measure ($TU_{E}^{I}$)“, proposed by Deng et al. in 2017, is an improved total uncertainty measure method based on the belief interval.

In Figure 1, the uncertainty calculated by Yang and Han’s measure, $TU_{E}^{I}$ and the weighted Hartley entropy show a downward trend with the increase of *n*, which was inconsistent with our intuition. However, the uncertainty calculated by the proposed methods in this paper and the remaining existing methods gradually increases with the increase of *n*, which is consistent with the results calculated by Shannon entropy. Therefore, the proposed methods in this paper are effective when BPA is a Bayesian mass function.

### 4.2. Example 2

**Figure 2:**
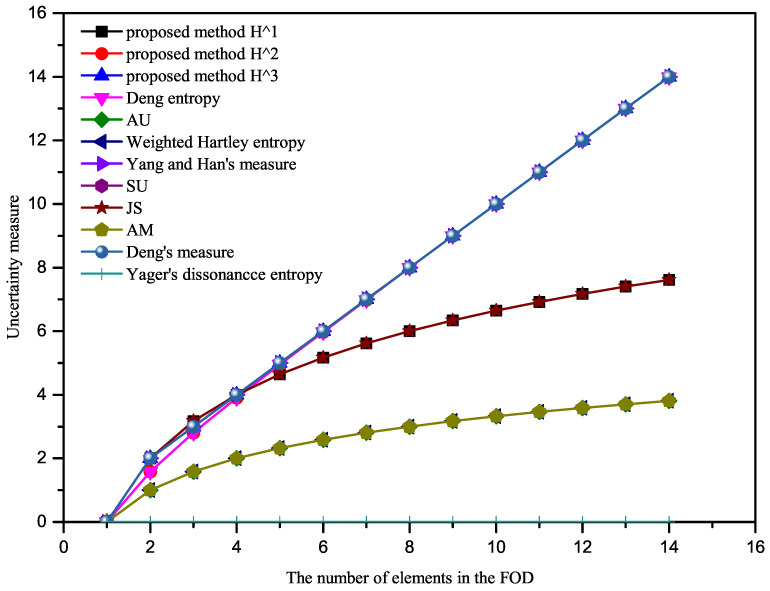
Comparison results of Example 2.

Let the FOD be $X=\left\{x_{1},x_{2},\cdots ,x_{n}\right\}$. We also give a BPA as $m\left(\left\{x_{1},\cdots ,x_{n}\right\}\right)=1$. When *n* increases from 1 to 14, the uncertainty measure of BPAs based on the proposed method and other existing methods are shown in Table 1. In addition, in order to visualize the change of uncertain measurement results with *n*, the uncertainty measurement results of different methods are given in Figure 2.

As shown in Figure 2, it is obvious that the uncertainty degree measured by the Yager’s dissonance entropy is always 0. Intuitively, however, the uncertainty of this BPA should increase as *n* increases. Therefore, Yager’s dissonance entropy will obtain wrong results when *m* is a vacuous BPA in this example.

The uncertainty measure results obtained by AU, weighted Hartley entropy, and AM are the same. This is because when BPA is a vacuous BPA, the three methods give us the same result $\log_{2}\left|n\right|$. The degree of uncertainty obtained by these three methods increases with the increase of *n*, which is consistent with expectations. Similarly, the degree of uncertainty calculated based on the methods proposed in this paper ($H^{1}$, $H^{2}$, $H^{3}$), Deng’s entropy, SU, JS, Yang and Han’s measure, and $TU_{E}^{I}$ also increases with the increase of *n*. Additionally, the growth trend of the proposed method $H^{2}$ in this paper is basically the same as that of the Deng entropy. This is because for a vacuous BPA, $m\left(A\right)=Pl\left(A\right)$. Hence, the two methods have the same function form. The proposed method $H^{3}$ and SU, Yang and Han’s measure, $TU_{E}^{I}$ also have the same growth trend. However, we think that when the change trend is consistent with the theoretical connotation of uncertainty, it can be considered as a reasonable and effective measure method. Hence, the proposed methods are all effective when BPA is a vacuous BPA.

For this example, the proposed method $H^{2}$ gave the same results as the Deng entropy.

### 4.3. Example 3

Let $X=\left\{a,b,c,d\right\}$ be the FOD. We give two BPAs as follows.

$$\begin{array}{c} m_{1}:\;m_{1}\left(\left\{a\right\}\right)=\frac{1}{5},\;m_{1}\left(\left\{b\right\}\right)=\frac{1}{5},\;m_{1}\left(\left\{a,b\right\}\right)=\frac{3}{5}\\ m_{2}:\;m_{2}\left(\left\{a\right\}\right)=\frac{1}{5},\;m_{2}\left(\left\{b\right\}\right)=\frac{1}{5},\;m_{2}\left(\left\{c,d\right\}\right)=\frac{3}{5} \end{array}.$$

For $m_{1}$,

$$Pl_{m_{1}}\left(\left\{a\right\}\right)=\frac{4}{5},\;Pl_{m_{1}}\left(\left\{b\right\}\right)=\frac{4}{5},\;Pl_{m_{1}}\left(\left\{a,b\right\}\right)=1,$$

for $m_{2}$,

$$Pl_{m_{2}}\left(\left\{a\right\}\right)=\frac{1}{5},\;Pl_{m_{2}}\left(\left\{b\right\}\right)=\frac{1}{5},\;Pl_{m_{2}}\left(\left\{c,d\right\}\right)=\frac{3}{5},$$

then the uncertainty based on the proposed method are:

$$\begin{array}{ccc} H^{1}\left(m_{1}\right) & = & \frac{1}{5}\cdot \log_{2}\frac{2^{2\cdot \log_{2}1}}{4/5}+\frac{1}{5}\cdot \log_{2}\frac{2^{2\cdot \log_{2}1}}{4/5}+\frac{3}{5}\cdot \log_{2}\frac{2^{2\cdot \log_{2}2}}{1}\\ & = & 2\times \frac{1}{5}\cdot \log_{2}\frac{1}{4/5}+\frac{3}{5}\times 2=1.3288. \end{array}$$

$$\begin{array}{ccc} H^{2}\left(m_{1}\right) & = & \frac{1}{5}\cdot \log_{2}\frac{2^{\log_{2}\left(2^{1}-1\right)}}{4/5}+\frac{1}{5}\cdot \log_{2}\frac{2^{\log_{2}\left(2^{1}-1\right)}}{4/5}+\frac{3}{5}\cdot \log_{2}\frac{2^{\log_{2}\left(2^{2}-1\right)}}{1}\\ & = & 2\times \frac{1}{5}\cdot \log_{2}\frac{1}{4/5}+\frac{3}{5}\times \log_{2}3=1.0797. \end{array}$$

$$\begin{array}{ccc} H^{3}\left(m_{1}\right) & = & \frac{1}{5}\cdot \log_{2}\frac{2^{0}}{4/5}+\frac{1}{5}\cdot \log_{2}\frac{2^{0}}{4/5}+\frac{3}{5}\cdot \log_{2}\frac{2^{2}}{1}\\ & = & 2\times \frac{1}{5}\cdot \log_{2}\frac{1}{4/5}+\frac{3}{5}\times \log_{2}4=1.3288. \end{array}$$

$$\begin{array}{ccc} H^{1}\left(m_{2}\right) & = & \frac{1}{5}\cdot \log_{2}\frac{2^{2\cdot \log_{2}1}}{1/5}+\frac{1}{5}\cdot \log_{2}\frac{2^{2\cdot \log_{2}1}}{1/5}+\frac{3}{5}\cdot \log_{2}\frac{2^{2\cdot \log_{2}2}}{3/5}\\ & = & 2\times \frac{1}{5}\cdot \log_{2}\frac{1}{1/5}+\frac{3}{5}\times \log_{2}\frac{4}{3/5}=2.5710. \end{array}$$

$$\begin{array}{ccc} H^{2}\left(m_{2}\right) & = & \frac{1}{5}\cdot \log_{2}\frac{2^{\log_{2}\left(2^{1}-1\right)}}{1/5}+\frac{1}{5}\cdot \log_{2}\frac{2^{\log_{2}\left(2^{1}-1\right)}}{1/5}+\frac{3}{5}\cdot \log_{2}\frac{2^{\log_{2}\left(2^{2}-1\right)}}{3/5}\\ & = & 2\times \frac{1}{5}\cdot \log_{2}\frac{1}{1/5}+\frac{3}{5}\times \log_{2}\frac{3}{3/5}=2.3219. \end{array}$$

$$\begin{array}{ccc} H^{3}\left(m_{2}\right) & = & \frac{1}{5}\cdot \log_{2}\frac{2^{0}}{1/5}+\frac{1}{5}\cdot \log_{2}\frac{2^{0}}{1/5}+\frac{3}{5}\cdot \log_{2}\frac{2^{2}}{3/5}\\ & = & 2\times \frac{1}{5}\cdot \log_{2}\frac{1}{1/5}+\frac{3}{5}\times \log_{2}\frac{4}{3/5}=2.5710. \end{array}$$

In addition, the uncertainty measured by other methods, as shown in Table 2.

Obviously, owing to differences in the focal elements in these BPAs, the uncertainty degree of $m_{1}$ and $m_{2}$ are different, though the values of the two BPAs are the same, and $H\left(m_{1}\right)$ should be less than $H\left(m_{2}\right)$. However, the results obtained by the Deng entropy and weighted Hartley entropy are the same for $m_{1}$ and $m_{2}$. The results obtained by other methods are as expected. However, Yager’s dissonance entropy method did not consider the non-specificity measure. The methods proposed in this paper obtain reasonable results and consider the total uncertainty. Therefore, when the focal elements are different but the BPA values are the same, the proposed method in this paper can effectively measure the degree of uncertainty.

### 4.4. Example 4

Let $X=\left\{a,b,c,d\right\}$ be a FOD. Two BPAs defined on the FOD are given as follows. There is an intersection relationship between propositions.

$$\begin{array}{c} m_{1}:\;m_{1}\left(\left\{a,b\right\}\right)=0.4,\;m_{1}\left(\left\{c,d\right\}\right)=0.6\\ m_{2}:\;m_{2}\left(\left\{a,c\right\}\right)=0.4,\;m_{2}\left(\left\{b,c\right\}\right)=0.6 \end{array}$$

The uncertainties are measured by different methods, as shown in Table 3.

For the body of evidence (BOE) $m_{1}$, the intersection between the two propositions a,b and c,d is empty. For BOE $m_{2}$, the intersection between the two propositions a,b and c,d is a single element *c*. However, the values of BPAs are the same. Based on the above analysis, the uncertainties of two BOEs are obviously different. However, according to Table 3, the results are the same for $m_{1}$ and $m_{2}$, measured by Deng entropy, AU, weighted Hartley entropy, Yang and Han’s measure, and Deng’s measure. Besides, the uncertainty of BOE $m_{2}$ calculated by Yager’s dissonance entropy is 0. This result is clearly wrong. This is because there is an intersection between two propositions of $m_{2}$, and Yager’s dissonance entropy eliminates the distinction between the two propositions when calculating a plausibility function. On the contrary, the three methods we propose can all distinguish the uncertainty difference between the two BOEs. Therefore, the proposed method can effectively distinguish the uncertainty when there is an intersection relationship between propositions.

### 4.5. Example 5

Let $X=\left\{1,2,\cdots ,15\right\}$ be a FOD with 15 elements. A BPA defined on the FOD is:

$$m\left(3,4,5\right)=0.05,\;m\left(7\right)=0.05,\;m\left(A\right)=0.8,\;m\left(X\right)=0.1,$$

where *A* is a variable subset of *X*, with the number of single elements changing from 1 to 14. This example was adopted from [53].

**Figure 3:**
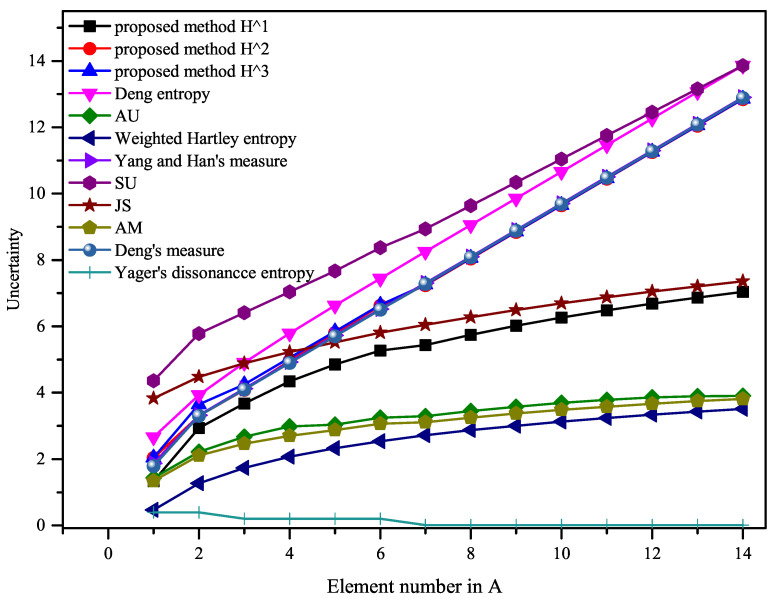
Comparison results of Example 5.

The results are shown in Table 4 and Table 5, and Figure 3. Table 4 shows changes in the number of elements in *S* from 1 to 7, and Table 5 shows changes in the number of elements in *A* from 8 to 14. All the results are plotted in Figure 3.

As shown in Figure 3, with the increase of the number of elements in *A*, the uncertainty calculated by Yager’s dissonance entropy shows a downward trend, which is inconsistent with the connotation of uncertainty. The reason is that when the number of elements in *A* gradually increases, it gradually intersects with other propositions, and Yager’s entropy does not show this difference. This suggests that Yager’s entropy does not correctly measure the uncertainty of the evidence in this example.

**Figure 4:**
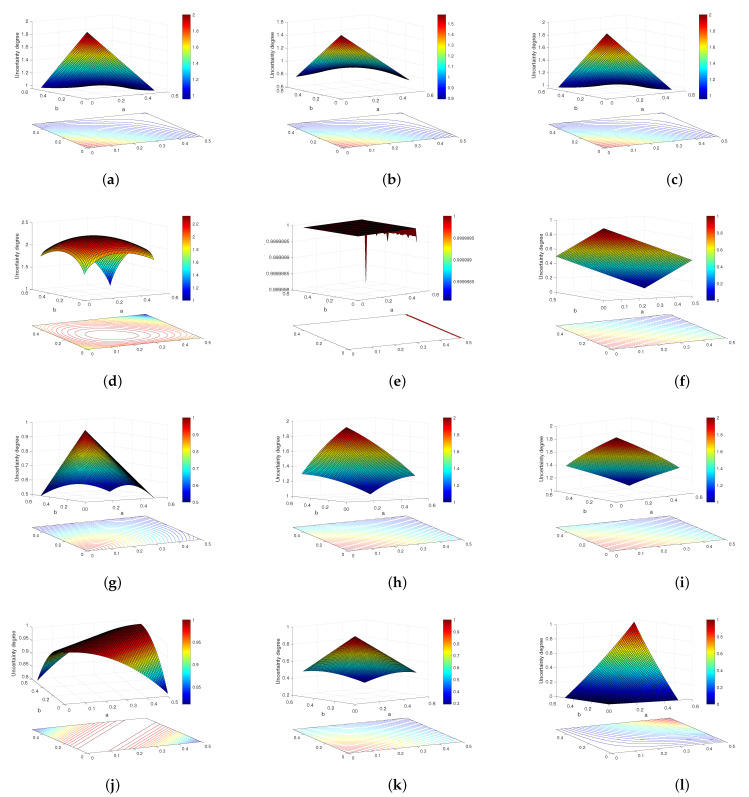
Comparison results of Example 7. (**a**) The proposed method $H^{1}$; (**b**) the proposed method $H^{2}$; (**c**) the proposed method $H^{3}$; (**d**) Deng entropy; (**e**) AU; (**f**) weighted Hartley entropy; (**g**) Yang and Han’s measure; (**h**) SU; (i)JS; (j) AM; (**k**) Deng’s measure($TU_{E}^{I}$); (l) Yager’s dissonance entropy.

From a “common sense” point of view, the uncertainty of BPA increases as the number of elements increases in *A*. Additionally, other methods except Yager’s dissonance entropy show an increasing trend on the whole. The corresponding values can be found in Table 4 and Table 5. However, it should be noted that when *A* changes from {1,2} to {1,2,3}, *A* begins to intersect with the proposition {3,4,5}. Therefore, the change of the uncertainty should be slightly less than the change of *A* from {1} to {1,2}. From Figure 4, it can be obtained that the proposed methods in this paper present this change. As for the uncertainty measure of BOEs, as far as we know, there is no reasonable evaluation index at present, and it is not certain that the greater the uncertainty, the better. Nevertheless, when its change trend is consistent with the theoretical connotation of uncertainty, it can be considered as a reasonable and effective measurement method.

### 4.6. Example 6

Let $X=\left\{\theta_{1},\theta_{2}\right\}$ be a FOD with two elements. Additionally, we give a BPA as:

$$m\left(\left\{\theta_{1}\right\}\right)=a,\;m\left(\left\{\theta_{2}\right\}\right)=b,\;m\left(\left\{\theta_{1},\theta_{2}\right\}\right)=1-a-b,$$

where $a,b\in \left[0,0.5\right]$. This example is adopted from [53]. Here, we calculate the uncertainty values based on the proposed methods and some existing methods with the changes of *a* and *b*. The results are shown in Figure 4.

For the proposed methods $H^{1}$, $H^{2}$, and $H^{3}$, Yang and Han’s measure, weighted Hartley entropy, SU, JS, and Deng’s measure ($TU_{E}^{I}$), it can be found that the maximum uncertainty is obtained when $m\left(X\right)=1$, which is consistent with the property of maximum entropy. In addition, as the value of $m\left(X\right)$ decreases, the uncertainty of BOE decreases gradually. As for Deng entropy, according to [26], its maximum uncertainty is obtained at $m\left(\theta_{1}\right)=\frac{2^{\left|\theta_{1}\right|}-1}{\left(2^{\left|\theta_{1}\right|}-1\right)+\left(2^{\left|\theta_{2}\right|}-1\right)+\left(2^{\left|\left\{\theta_{1},\theta_{2}\right\}\right|}-1\right)}=0.2$, $m\left(\theta_{2}\right)=0.2$, and $m\left(X\right)=0.6$, which is consistent with Figure 4d and does not satisfy the maximum entropy property. For Yager’s dissonance entropy, the maximum uncertainty is obtained at $m\left(\theta_{1}\right)=0.5,m\left(\theta_{2}\right)=0.5,m\left(X\right)=0$. As the value of $m\left(X\right)$ decreases, the uncertainty also increases, which is counter-intuitive. Hence, in this example, Yager’s dissonance entropy fails to measure the uncertainty of BOEs. Besides, for the method of AU, it obviously fails to measure the uncertainty of BOEs in this example.

The above numerical examples are analyzed and summarized. For Example 1, since we give a Bayesian mass function, the uncertainty of the Bayesian BPA is proportional to the number of elements *n* in FOD based on the proposed method. It can also be understood from the concept of entropy that with the increase of the elements number of FOD, the “chaos” degree of information in this example also increases. The results obtained by all methods are consistent with this understanding, except weighted Hartley entropy, and two different “measures” of Yang and Han, as well as Deng. For Example 2, we give a vacuous BPA. Obviously, as the number of elements in the FOD increases, the disorder of the system increases. This is true for all methods except Yager’s dissonance entropy. It is because Yager’s dissonance entropy only measures dissonance, not non-specificity. For Examples 3 and 4, the two BPAs given in each example are assigned the same belief value, but with different propositions. Because the uncertainty of a BPA is related to its belief value and proposition, the uncertainty is obviously different when the propositions are completely disjointed and partially intersected. Example 5 further illustrates the problem. In this example, the degree of intersection between the different propositions of the BPA changes gradually, that is, the belief values for elements in the FOD change gradually. Therefore, the degree of confusion in the system changes gradually, and the results of the uncertainty measure change accordingly. However, overall, as the number of elements in *A* increases, the confusion of BPA to the system should also increase. For Example 6, different belief values are assigned to BPA, but the propositions are the same. Obviously, when $m\left(\left\{\theta_{1},\theta_{2}\right\}\right)=1$, the system is in a completely unknown state, so the uncertainty should be at the maximum, which is the maximum entropy.

The proposed method in this paper can effectively measure the uncertainty of BOEs in the above examples. However, for the non-specificity measure of BPA, $Q_{S}$ function is determined based on the maximum entropy of three existing uncertainty measures. Actually, there are many other entropies which can be considered, such as info-entropy [32]. This is a good guide for our future research direction.

## 5. Application

**Figure 5:**
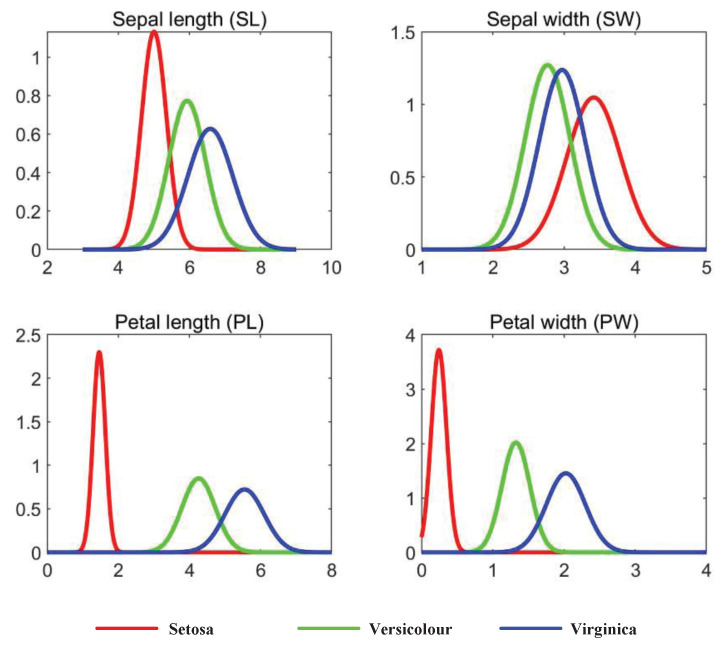
Probability density functions (PDFs) of different features of samples in the Iris dataset.

In this section, feature evaluation is performed with the Iris dataset to further verify the rationality of the proposed uncertainty measure. In the Iris dataset, three types of iris plants are surveyed, including “Setosa“, “Versicolour“, and “Virginica“. Besides, sepal length (SL), sepal width (SW), petal length (PL), and petal width (PW) are taken as four features. With respect to each iris class, each feature of instances is a Gaussian distribution with different standard deviations and means, as shown in Table 6 and Figure 5.

As shown in Figure 5, intuitively, PL has the best class discriminability, which is attributed to the best separation of Gaussian probability density functions (PDFs) of the three iris types, while the PDFs of the three iris types in SW almost overlap. Thus, the class discriminability of SW is the worst.

In addition, the method proposed in [54] is utilized to quantify the discriminability of different fault features, which is as shown below.

(20) $$J=\frac{tr\left(S_{w}\right)}{tr\left(S_{b}\right)},$$

where tr is the trace of a matrix, and $S_{w}$ and $S_{b}$ are the within-types scatter matrix and between-types scatter matrix, respectively.

(21) $$\left\{\begin{array}{c} S_{w}=\sum_{i=1}^{C}P\left(C_{i}\right)E\left[\left(X-\frac{1}{N_{i}}\sum_{X\in C_{i}}X\right){\left(X-\frac{1}{N_{i}}\sum_{X\in C_{i}}X\right)}^{T}\right]\\ S_{b}=\sum_{i=1}^{C}P\left(C_{i}\right)\left(\frac{1}{N_{i}}\sum_{X\in C_{i}}X-M\right){\left(\frac{1}{N_{i}}\sum_{X\in C_{i}}X-M\right)}^{T} \end{array}\right.,$$

with

(22) $$M=\frac{1}{C}\sum_{i=1}^{C}\left(\frac{1}{N_{i}}\sum_{X\in C_{i}}X\right),$$

where *X* is a feature vector of a sample and *M* is the mean of all fault types’ centroids.

The smaller the value of *J*, the better the discriminability of the corresponding fault feature. For the fault features, the *J* values are

$$J\left(SL\right)=0.6163,J\left(SW\right)=1.5518,J\left(PL\right)=0.0623,J\left(PW\right)=0.0766.$$

The above results are the same as those intuitively obtained in Figure 5. The rank of the four fault features is PL≻PW≻SL≻SW.

Now, we turn to using uncertainty measures for feature evaluation, including weighted Hartley entropy, AU, Yang and Han’s measure, Deng’s measure, AM, JS, Deng entropy, SU, and the proposed method.

**Step1 (BPA generation).**  For different features in $\left\{SL,SW,PL,PW\right\}$, we generate the BPA corresponding to each sample in the fault dataset according to [55].
**Step2 (Uncertainty measure of BPAs).**  For each feature, calculate the uncertainty of each BPA on it by using all the above uncertainty measures.
**Step3 (Average uncertainty measure).**  Calculate the average uncertainty value on different features corresponding to different methods.

**Figure 6:**
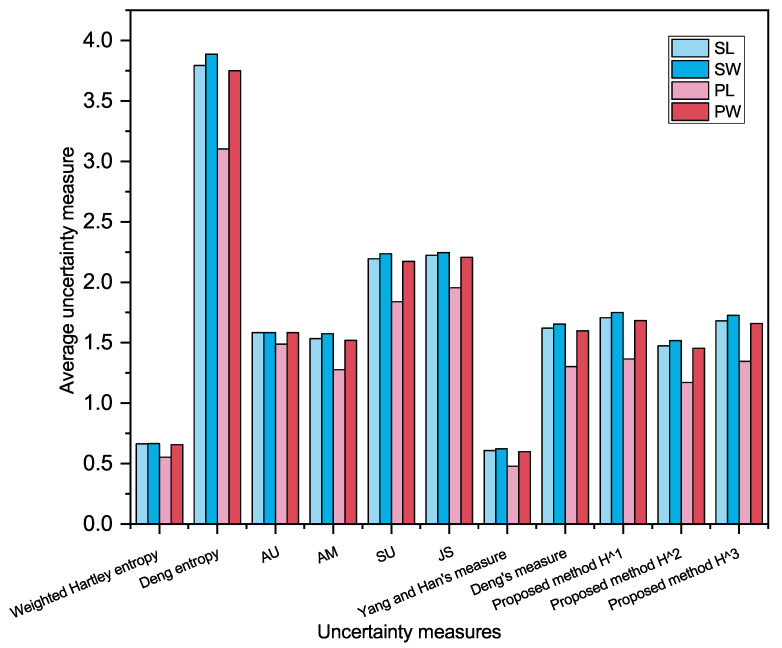
Average uncertainty of samples on each feature based on different uncertainty measures.

The results are shown in Table 7, and visually represented in the histogram, which are shown in Figure 6.

Features with smaller average uncertainty have better discriminability. It can be found in Table 7 and Figure 6 that for the proposed methods $H^{1}$, $H^{2}$ and $H^{3}$, the average uncertainty on feature PL is the smallest. The result indicates the feature PL has the best ability to distinguish the iris types. The ranking of the discrimination of the four features is PL≻PW≻SL≻SW, which is consistent with intuition obtained by Figure 5. The same result can be obtained by Deng entropy, AM, SU, JS, Yang and Han’s measure, and Deng’s measure except weighted Hartley entropy and AU. Therefore, the application has demonstrated the effectiveness of the proposed method.

## 6. Conclusions

In this work, we proposed a new total uncertainty measure from the perspective of maximum entropy requirement. The properties of the proposed method are analyzed, such as non-negativity, monotonicity, maximum entropy, and so on. Besides, we give three uncertainty measure functions for the body of evidences, and analyze the effectiveness and reasonableness of the proposed methods through several different numerical examples and an application. It can be seen from these examples that our methods are in general agreement with the connotation of uncertainty. Compared with Deng entropy, the proposed method can effectively measure the uncertainty of BPA when the propositions intersect with the same reliability value, and satisfy the maximum entropy property. In addition, it is not that the greater the uncertainty for a BPA, the better the measure. How do we evaluate uncertainty measure methods more rationally in DST? Is there a reasonable indicator system for the evaluation? Actually, we think that when we adapt the proposed method, and the uncertainty trend of the measured evidence is consistent with the theory, it can be considered as a reasonable and effective method. Our study provides the framework for further studies to assess the performance characteristics of uncertainty function. Our results are encouraging and should be validated in application areas, such as decision-making, fault diagnosis, target recognition, and many other areas. We will be devoted to the applications in depth in further work. Beyond that, a maximum entropy from the Parker and Jeynes argument showed that the entropy of the supermassive black hole at the centre of the Milky Way can account for the geometrical stability of the galaxy. We believe that this is a good guide for our future work about uncertainty measures.

## Figures and Tables

**Table 1:** The comparison between the proposed method and some existing methods in Example 2.

Uncertainty Measures	n=1	n=2	n=3	n=4	n=5	n=6	n=7	n=8	n=9	n=10	n=11	n=12	n=13	n=14
Deng entropy	0	1.5850	2.8074	3.9069	4.9542	5.9773	6.9887	7.9944	8.9972	9.9986	10.9993	11.9996	12.9998	13.9999
AU	0	1	1.5850	2	2.3219	2.5850	2.8074	3	3.1699	3.3219	3.4594	3.5850	3.7004	3.8074
Weighted Hartley entropy	0	1	1.5850	2	2.3219	2.5850	2.8074	3	3.1699	3.3219	3.4594	3.5850	3.7004	3.8074
Yang and Han’s measure	0	2	3	4	5	6	7	8	9	10	11	12	13	14
SU	0	2	3	4	5	6	7	8	9	10	11	12	13	14
JS	0	2	3.1699	4	4.6439	5.1699	5.6147	6	6.3399	6.6439	6.9189	7.1699	7.4009	7.6147
AM	0	1	1.5850	2	2.3219	2.5850	2.8074	3	3.1699	3.3219	3.4594	3.5850	3.7004	3.8074
Deng’s measure($TU_{E}^{I}$)	0	2	3	4	5	6	7	8	9	10	11	12	13	14
Yager’s dissonance entropy	0	0	0	0	0	0	0	0	0	0	0	0	0	0
Proposed method $H^{1}$	0	2	3.1699	4	4.6439	5.1699	5.6147	6	6.3399	6.6439	6.9189	7.1699	7.4009	7.6147
Proposed method $H^{2}$	0	1.5850	2.8074	3.9069	4.9542	5.9773	6.9887	7.9944	8.9972	9.9986	10.9993	11.9996	12.9998	13.9999
Proposed method $H^{3}$	0	2	3	4	5	6	7	8	9	10	11	12	13	14

**Table 2:** The uncertainty measured by other methods in Example 3.

Uncertainty Measures	$m_{1}$	$m_{2}$
Deng entropy	2.3219	2.3219
AU	1	1.9710
Weighted Hartley entropy	0.6	0.6
Yang and Han’s measure	0.4	0.4394
SU	1.6	2.5710
JS	1.6	2.4113
AM	1	1.9710
Deng’s measure ($TU_{E}^{I}$)	0.3586	0.3877
Yager’s dissonance entropy	0.1288	1.3710

**Table 3:** The uncertainty measured by different methods in Example 4.

Uncertainty Measures	$m_{1}$	$m_{2}$
Deng entropy	2.5559	2.5559
AU	1.9710	1.9710
Weighted Hartley entropy	1.0000	1.0000
Yang and Han’s measure	0.5000	0.5000
SU	2.9710	2.4855
JS	2.9710	2.4855
AM	1.9710	1.4855
Deng’s measure ($TU_{E}^{I}$)	0.5000	0.5000
Yager’s dissonance entropy	0.9710	0
Proposed method $H^{1}$	2.9710	2.0000
Proposed method $H^{2}$	2.5559	1.5850
Proposed method $H^{3}$	2.9710	2.0000

**Table 4:** The comparison between the proposed method and some existing methods in Example 5.

Uncertainty Measures	$A=\left\{1\right\}$	$A=\left\{1,2\right\}$	$A=\left\{1,2,3\right\}$	$A=\left\{1,2,3,4\right\}$	$A=\left\{1,2,\cdots ,5\right\}$	$A=\left\{1,2,\cdots ,6\right\}$	$A=\left\{1,2,\cdots ,7\right\}$
Deng entropy	2.6623	2.9303	4.9082	5.7878	6.6256	7.4441	8.2532
AU	1.4328	2.2180	2.6707	2.9821	3.0378	3.2447	3.2956
Weighted Hartley entropy	0.4699	1.2699	1.7379	2.0699	2.3275	2.5379	2.7158
Yang and Han’s measure	0.1246	0.2216	0.2749	0.3283	0.3816	0.4349	0.4867
SU	4.3583	5.7797	6.4096	7.0394	7.6693	8.3716	8.9394
JS	3.8322	4.4789	4.8870	5.2250	5.5200	5.8059	6.0425
AM	1.3461	2.1037	2.4623	2.7011	2.8762	3.0684	3.1083
Deng’s measure ($TU_{E}^{I}$ )	0.1195	0.2199	0.2732	0.3266	0.3799	0.4332	0.4853
Yager’s dissonance entropy	0.3953	0.3953	0.1997	0.1997	0.1997	0.1997	0.0074
Proposed method $H^{1}$	1.3352	2.9352	3.6756	4.3396	4.8547	5.2756	5.4390
Proposed method $H^{2}$	2.0357	3.3036	4.0860	4.9656	5.8035	6.6219	7.2387
Proposed method $H^{3}$	2.0453	3.6453	4.2497	5.0497	5.8497	6.6497	7.2574

**Table 5:** The comparison between the proposed method and some existing methods in Example 5.

Uncertainty Measures	$A=\left\{1,2,\cdots ,8\right\}$	$A=\left\{1,2,\cdots ,9\right\}$	$A=\left\{1,2,\cdots ,10\right\}$	$A=\left\{1,2,\cdots ,11\right\}$	$A=\left\{1,2,\cdots ,12\right\}$	$A=\left\{1,2,\cdots ,13\right\}$	$A=\left\{1,2,\cdots ,4\right\}$
Deng entropy	9.0578	9.8600	10.6612	11.4617	12.2620	13.0622	13.8622
AU	3.4497	3.5796	3.6909	3.7824	3.8538	3.8986	3.9069
Weighted Hartley entropy	2.8699	3.0059	3.1275	3.2375	3.3379	3.4303	3.5158
Yang and Han’s measure	0.5400	0.5933	0.6467	0.7000	0.7533	0.8067	0.8600
SU	9.6417	10.3440	11.0463	11.7486	12.4510	13.1533	13.8556
JS	6.2772	6.4921	6.6903	6.8743	7.0461	7.2071	7.3587
AM	3.2511	3.3747	3.4833	3.5797	3.6663	3.7446	3.8160
Deng’s measure ($TU_{E}^{I}$)	0.5386	0.5920	0.6453	0.6986	0.7520	0.8053	0.8586
Yager’s dissonance entropy	0.0074	0.0074	0.0074	0.0074	0.0074	0.0074	0.0074
Proposed method $H^{1}$	5.7473	6.0192	6.2624	6.4824	6.6832	6.8680	7.0390
Proposed method $H^{2}$	8.0432	8.8455	9.6466	10.4472	11.2475	12.0476	12.8477
Proposed method $H^{3}$	8.0574	8.8574	9.6574	10.4574	11.2574	12.0574	12.8574

**Table 6:** The mean value and standard deviation value for features.

Features		Setosa	Versicolour	Virginica
SL	Mean	5.0060	5.9360	6.5880
	Standard deviation	0.3525	0.5162	0.6359
SW	Mean	3.4180	2.7700	2.9740
	Standard deviation	0.3810	0.3138	0.3225
PL	Mean	1.4640	4.2600	5.5520
	Standard deviation	0.1735	0.4699	0.5519
PW	Mean	0.2440	1.3260	2.0260
	Standard deviation	0.1072	0.1978	0.2747

**Table 7:** The average uncertainty for different features.

Uncertainty Measures	SL	SW	PL	PW
Weighted Hartley entropy	0.6642	0.6660	0.5540	0.6556
Deng entropy	3.7394	3.8863	3.1025	3.7501
AU	1.5850	1.5850	1.4869	1.5850
AM	1.5333	1.5756	1.2764	1.5183
SU	2.1950	2.2364	1.8396	2.1723
JS	2.2226	2.2463	1.9536	2.2068
Yang and Han’s measure	0.6072	0.6227	0.4787	0.5986
Deng’s measure	1.6208	1.6532	1.3023	1.5981
Proposed method $H^{1}$	1.7057	1.7509	1.3665	1.6833
Proposed method $H^{2}$	1.4729	1.5178	1.1709	1.4542
Proposed method $H^{3}$	1.6810	1.7260	1.3467	1.6586

## Data Availability

Not applicable.

## Abbreviations

The following abbreviations are used in this manuscript:

PT	probability theory
DST	Dempster-Shafer evidence theory
FOD	Frame of Discernment
BPA	basic probability assignment
BOE	body of evidence
